# High Temperature Triggers Systemic Necrosis and Reveals PRSV as the Primary Cause of “‘Mazi’ Watermelon”

**DOI:** 10.3390/ijms27219895

**Published:** 2026-11-04

**Authors:** Yaoxing Zang, Liming Liu, Pengyang Dou, Zhiling Liang, Yanfei Geng, Jingxin Shi, Boyang Hou, Baoshan Kang

**Affiliations:** 1 Zhongyuan Research Center, Chinese Academy of Agricultural Sciences, Xinxiang 453500, China; 2 Henan Key Laboratory of Fruit and Cucurbit Biology, Zhengzhou Fruit Research Institute, Chinese Academy of Agricultural Sciences, Zhengzhou 450009, China; 3 College of Horticulture, Henan Agriculture University, Zhengzhou 450046, China

**Keywords:** watermelon, necrosis, potyvirus, temperature, Mazi

## Abstract

Huaping County, Lijiang City, Yunnan Province, is a key production area for early-season winter watermelons. In recent years, watermelon fruits in this region have exhibited abnormal symptoms, such as protrusions and necrotic lesions on the surface, which has significantly reduced their commercial value; such fruits are known locally as “’Mazi’ watermelon”. Field symptoms included leaf mosaic, necrosis, malformation, and stem cracking, as well as epidermal protrusions and clustered necrosis. To identify the causal agents, typical diseased samples were collected from Xinzhuang Village, Huaping County, in April 2026. Systematic pathogen identification was conducted using tissue isolation, plate coating, and small RNA-seq techniques to detect fungal, bacterial, and viral pathogens. No pathogenic fungi or bacteria were isolated; however, small RNA sequencing identified the presence of papaya ringspot virus (PRSV) and zucchini tigré mosaic virus (ZTMV) in diseased plants. RT-PCR further confirmed mixed infection of PRSV and ZTMV in one sample and PRSV alone in another. Artificial inoculation experiments demonstrated that both viruses could induce mosaic and malformation symptoms in watermelon leaves, but disease manifestation was temperature-dependent. PRSV caused systemic necrosis under high temperatures (>28 °C), while ZTMV triggered mild necrosis at low temperatures (<25 °C). In greenhouse trials (22~35 °C), PRSV-inoculated plants developed stem cracking, fruit protrusions, and necrotic lesions, matching field “Mazi watermelon” symptoms. In conclusion, PRSV has been identified as the primary pathogen responsible for the “Mazi watermelon” symptoms in Huaping, and high temperature is a critical environmental factor that triggers systemic necrosis and severe fruit symptoms.

## 1. Introduction

Huaping County is, located in the dry-hot valley region of the Jinsha River in Lijiang, Yunnan Province, and is characterized by a typical subtropical climate with annual sunshine exceeding 2500 h and an average temperature of 19.2 °C. These unique climatic conditions provide an excellent natural environment for vegetable cultivation in early spring, and watermelon production has become important to local plateau-specific agriculture. By 2025, the cultivated area of watermelon reached 16,000 mu (approximately 1067 hectares), generating an annual output value exceeding 100 million yuan, significantly boosting farmers’ income. The primary cultivation method involves early-spring grafted open-field planting, which enhances yield and fruit quality through rootstock resistance and optimized agronomic practices. However, in recent years, a disease manifesting as raised protrusions, brown spots, or patchy blemishes on the fruit surface, locally known as “’Mazi’ watermelon”, has become prevalent in Huaping. This disease reduces marketable fruit yield by 20–50%, with severe cases resulting in yield reductions of up to 80%. It has persistently threatened the healthy development of the local watermelon industry for many years. Due to the complexity of pathogens in the field, similar symptoms can be caused by fungal, e.g., *Alternaria* spp. [1,2], *Cercospora citrullina* [3], bacterial, e.g., *Acidovorax citrulli* [4], or viral, e.g., melon necrotic spot virus [5] infections. However, no systematic pathogen identification has been reported to date. Clarifying the causative agents and epidemic patterns of “Mazi watermelon” is critical for developing effective control strategies and ensuring sustainable industry development. To address this gap, we collected symptomatic samples (leaves, vines, and fruits) from Huaping, and employed complementary approaches to identify the pathogens, including morphological characterization, molecular assays, and pathogenicity tests.

## 2. Results

### 2.1. Field Symptoms on Watermelon

**Figure 1:**
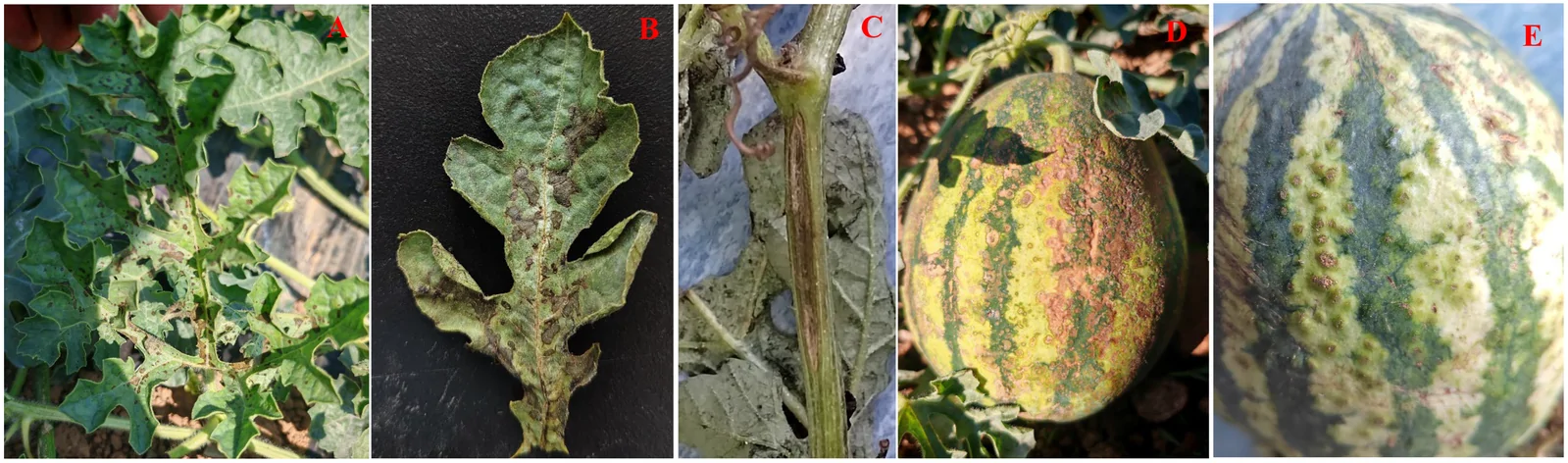
Field symptoms of “’Mazi’ watermelon”. (**A**,**B**): Necrotic lesions on leaves; (**C**): Stem cracking; (**D**,**E**): necrosis lesions and protrusions on fruit surfaces.

Field diseased plants exhibited brown spots on older leaves, while new leaves showed mosaic, crinkling, or malformation. Variable degree of longitudinal cracking of was observed locally on stems. The fruit epidermis was densely covered with raised, nearly circular spots, which coalesced into patches in some areas (Figure 1). Severe aphid infestations were observed in the fields. Based on the symptoms and vector presence, viral infection was preliminarily suspected; however, given the complex field environment, mixed infection with other pathogens could not be ruled out.

### 2.2. Fungal Pathogen Isolation and Identification

**Figure 2:**
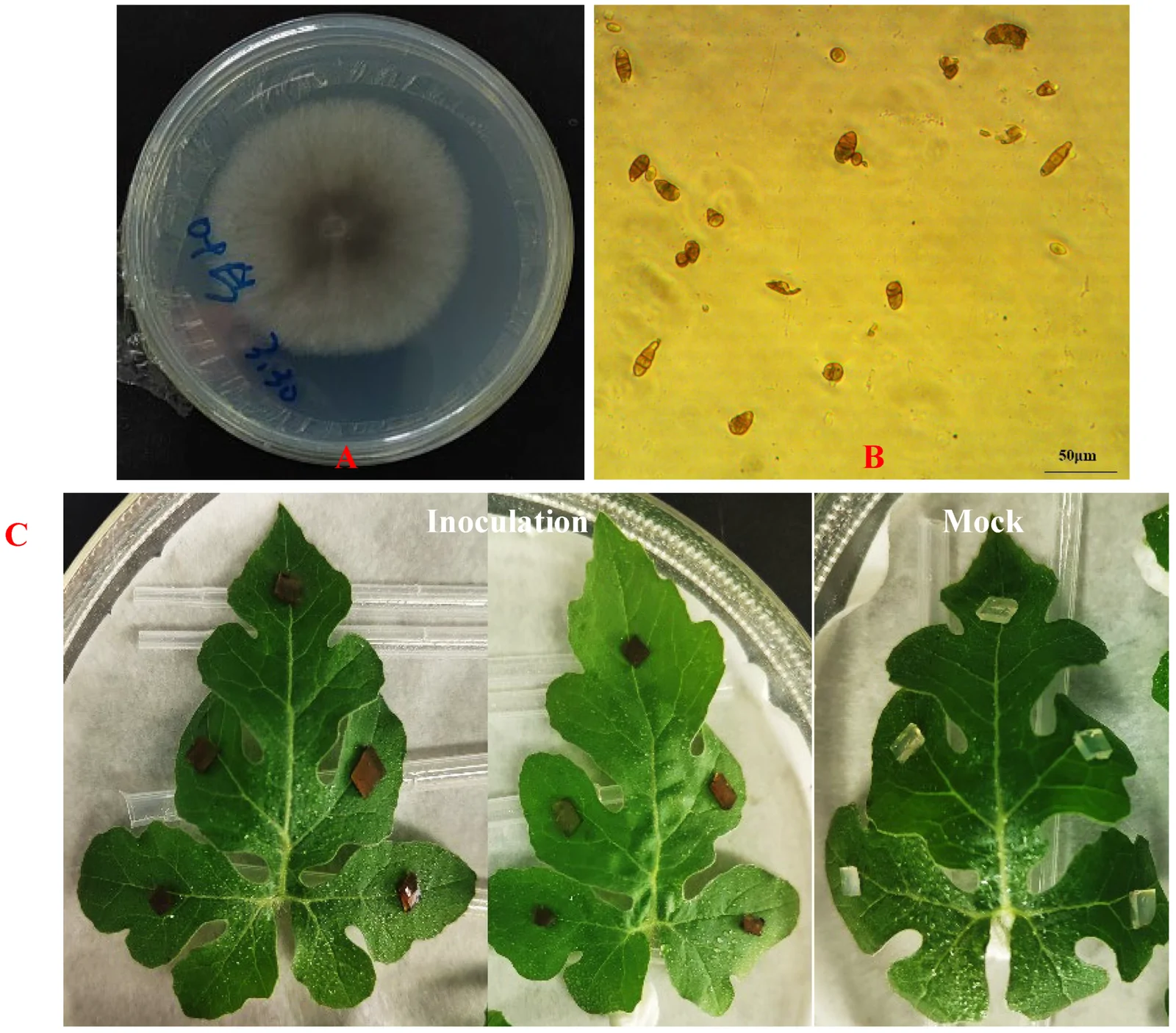
Fungal isolation and pathogenicity assay. (**A**), Colony morphology; (**B**), Spore morphology; (**C**), No symptoms on inoculated healthy leaves.

One fungal strain was isolated from leaf lesion tissues, purified to obtain morphologically consistent colonies. The colony morphology and spore morphology are shown in Figure 2A and Figure 2B, respectively. The fungus was identified as *Alternaria* sp. based on ITS sequencing. In vitro inoculation of mycelial plugs onto healthy watermelon leaves resulted in no lesions at the inoculation sites after 5 days (Figure 2C). No fungi were isolated from the stem and fruit lesion tissues. This indicates that the isolated fungus was not the primary pathogen causing the “Mazi” watermelon symptoms.

### 2.3. Bacterial Pathogen Isolation and Identification

Suspensions from fruit lesion tissues, diluted 5-fold and 50-fold, were spread on LB plates. No colonies were observed after incubation at 28 °C for 5 days. A few single colonies were isolated from leaf and stem tissues. Ten colonies were randomly selected for 16S rDNA amplification and sequencing. The BLASTn (National Center for Biotechnology Information, Bethesda, MD, USA, https://blast.ncbi.nlm.nih.gov, accessed on 4 October 2026) analysis of the sequence revealed that all 10 colonies represented distinct species: *Pseudomonas entomophila*, *Pseudomonas laurentiana*, *Pseudomonas* sp., *Methylorubrum aminovorans*, *Kosakonia pseudosacchari*, *Chryseobacterium lactis*, *Bacillus cereus*, *Acinetobacter baumannii*, *Paenibacillus* sp., and *Klebsiella* sp. Pathogenicity assays showed that none of these strains induced symptoms on watermelon seedlings at 10 dpi, indicating they were epiphytes or endophytes rather than pathogenic agents.

### 2.4. Virus Identification and Phylogenetic Analysis

Small RNA sequencing yielded 27,924,604 clean reads after adapter removal and filtering of short and low-quality reads. The data revealed the presence of two viruses in the diseased plants: papaya ringspot virus (PRSV) and zucchini tigré mosaic virus (ZTMV), with 4,176,330 and 9,282,678 reads assigned to them, respectively. The assembled PRSV and ZTMV contigs covered 84% and 81% of their respective reference genomes. The majority of PRSV contigs were mapped to the genome sequences of PRSV-XM (KY933061) or PRSV-LD (PX505302) isolates. The ZTMV contigs mapped to the genome sequences of ZTMV-WL1(MK988417), ZTMV-KF17 (MK988415) or ZTMV-CX1 (MK988416) isolates. Primers for full-length amplification of PRSV and ZTMV were designed based on the genomic sequences of PRSV-XM and ZTMV-CX1 isolates, respectively. RT-PCR was performed on samples of XZW1 and XZW2. The results revealed that sample XZW1 contained only PRSV (designated PRSV-HP1), whereas sample XZW2 harbored a mixed infection of PRSV (PRSV-HP2, PZ862977) and ZTMV (ZTMV-HP, PZ862976) (Figure S1). BLAST alignment of the deduced amino acid sequences from the full-length sequences showed 91.6% similarity between the PRSV-HP1 and PRSV-HP2 isolates.

**Figure 3:**
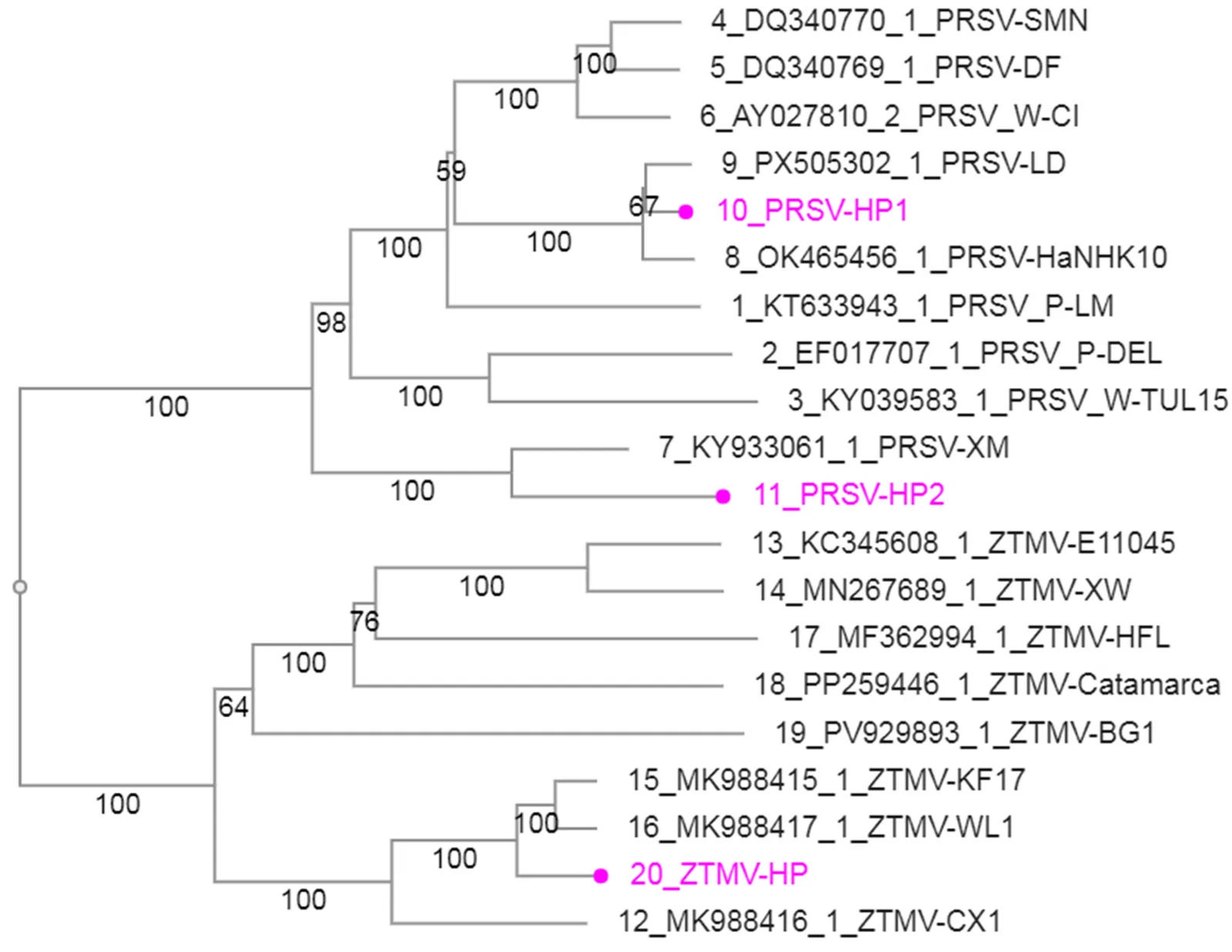
Phylogenetic trees based on alignment of the complete genome sequences of PRSV-HP1, HP2, ZTMV-HP and other isolates by using the neighbor-joining method with 1000 bootstrap replicates, and generated with the MAFFT v7 online server (https://mafft.cbrc.jp/alignment/server/, accessed on 23 September 2026) [6]. The numbers at the nodes represent bootstrap percentages (1000 replicates). The isolates in this study were highlighted in pink.

Phylogenetic analysis based on the full-length genomic sequences divided the tree into two major clades corresponding to PRSV and ZTMV (Figure 3). In the PRSV clade, PRSV-HP1 grouped with PRSV-LD (PX505302), and this pair was further grouped with PRSV-HaNHK10 (OK465456). PRSV-HP2 grouped with PRSV-XM (KY933061) as a distinct lineage, separate from the subclade containing PRSV-HP1. Within the ZTMV clade, ZTMV-KF17 (MK988415) and ZTMV-WL1 (MK988417) clustered together, and ZTMV-HP grouped with this pair, whereas ZTMV-CX1 (MK988416) formed the basal lineage of the clade. These results indicate that PRSV-HP1 and PRSV-HP2 belong to distinct evolutionary lineages within PRSV, and that ZTMV-HP is related to the ZTMV-KF17, ZTMV-WL1, and ZTMV-CX1 isolates.

### 2.5. High Temperature Induces Necrosis in PRSV-Infected Plants

**Figure 4:**
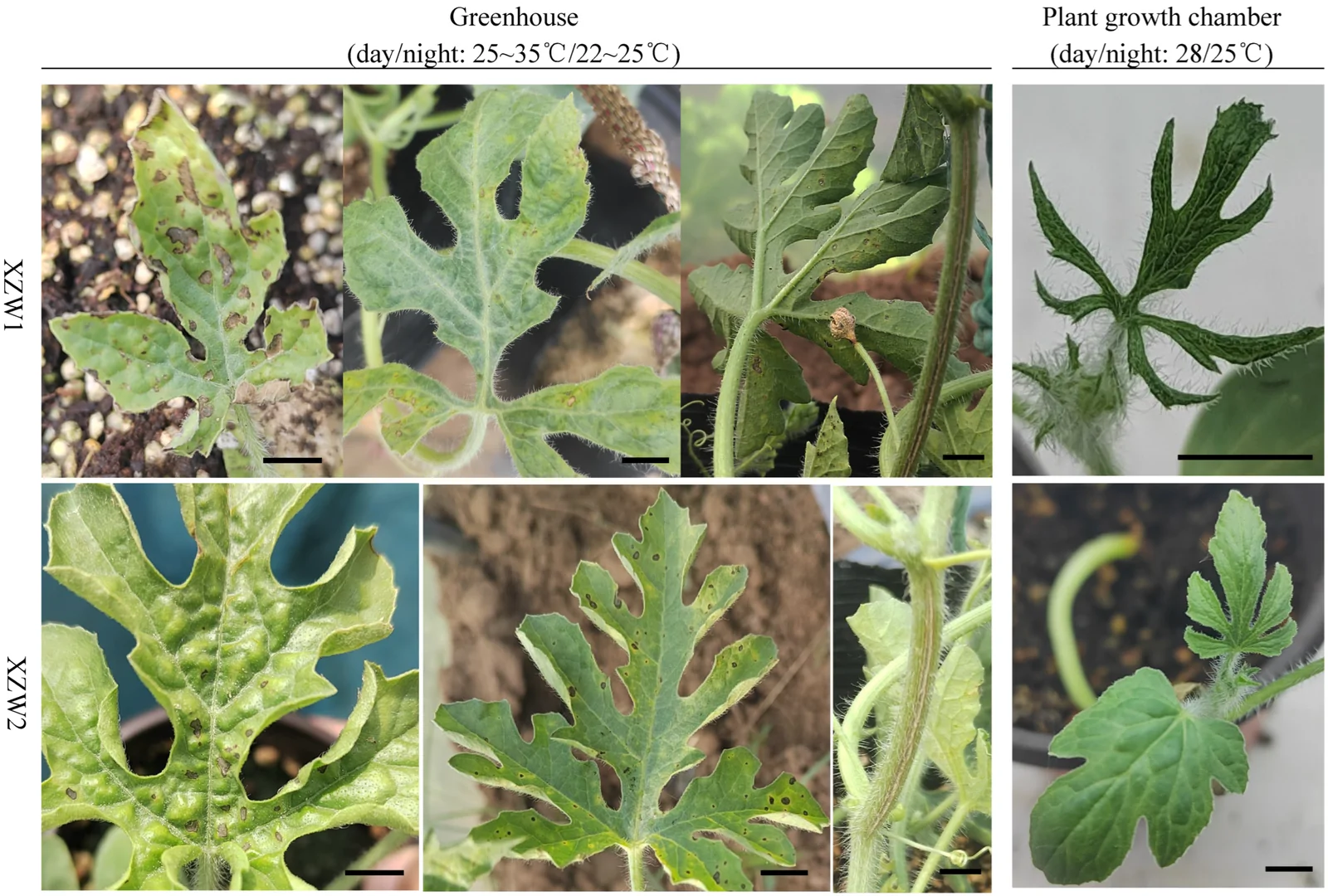
Symptoms of watermelon seedlings at 15 dpi with the sap of XZW1 and XZW2. Under greenhouse condition (22~35 °C), both inoculations caused mosaic, necrosis, and stem cracking in watermelon leaves at 15 dpi, while only leaf mosaic and malformation appeared at 28/25 °C. Bar: 1 cm.

To verify whether these viruses were the causal agents, healthy watermelon seedlings were mechanically inoculated with inoculum prepared from the epidermal lesion tissues of fruits from XZW1 and XZW2. Six days post-inoculation, new leaves began to exhibit mosaic symptoms, which gradually intensified, leading to leaf malformation and stunted plant growth. Plants placed in the greenhouse (daytime 25~35 °C) showed severe malformation of upper new leaves, necrotic lesions on old leaves, and stem cracking in some plants 15 days post-inoculation (Figure 4). These symptoms were highly consistent with those observed in “Mazi” watermelon plants in the field. In addition, RT-PCR using specific primers for the PRSV and ZTMV was performed on watermelon seedlings inoculated with diseased fruits. The results showed that the inoculated seedlings harbored the corresponding viruses matching their respective inocula. In brief, PRSV is the primary pathogen causing the “Mazi” watermelon symptoms in Huaping, with some diseased plants showing mixed infection with ZTMV.

**Figure 5:**
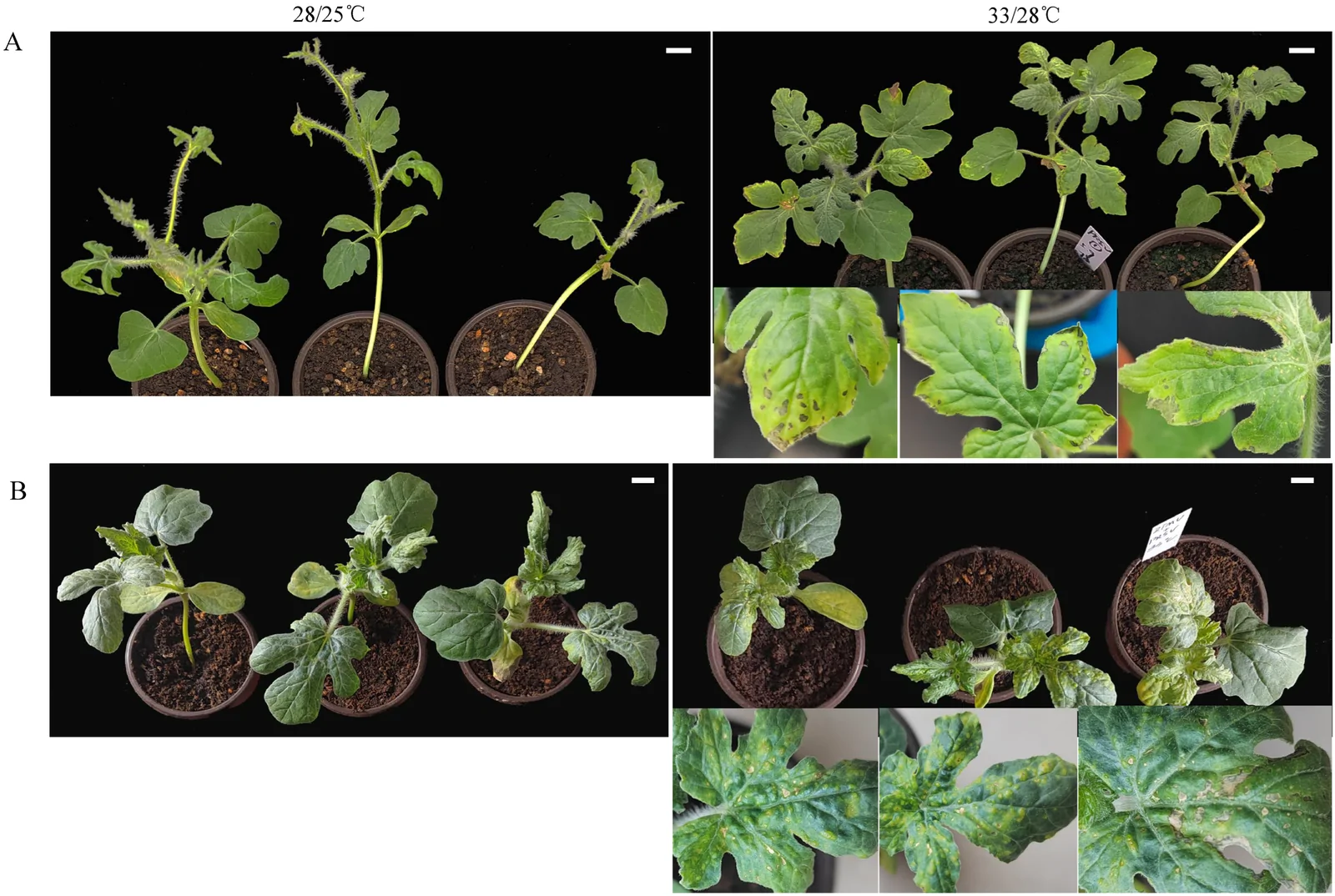

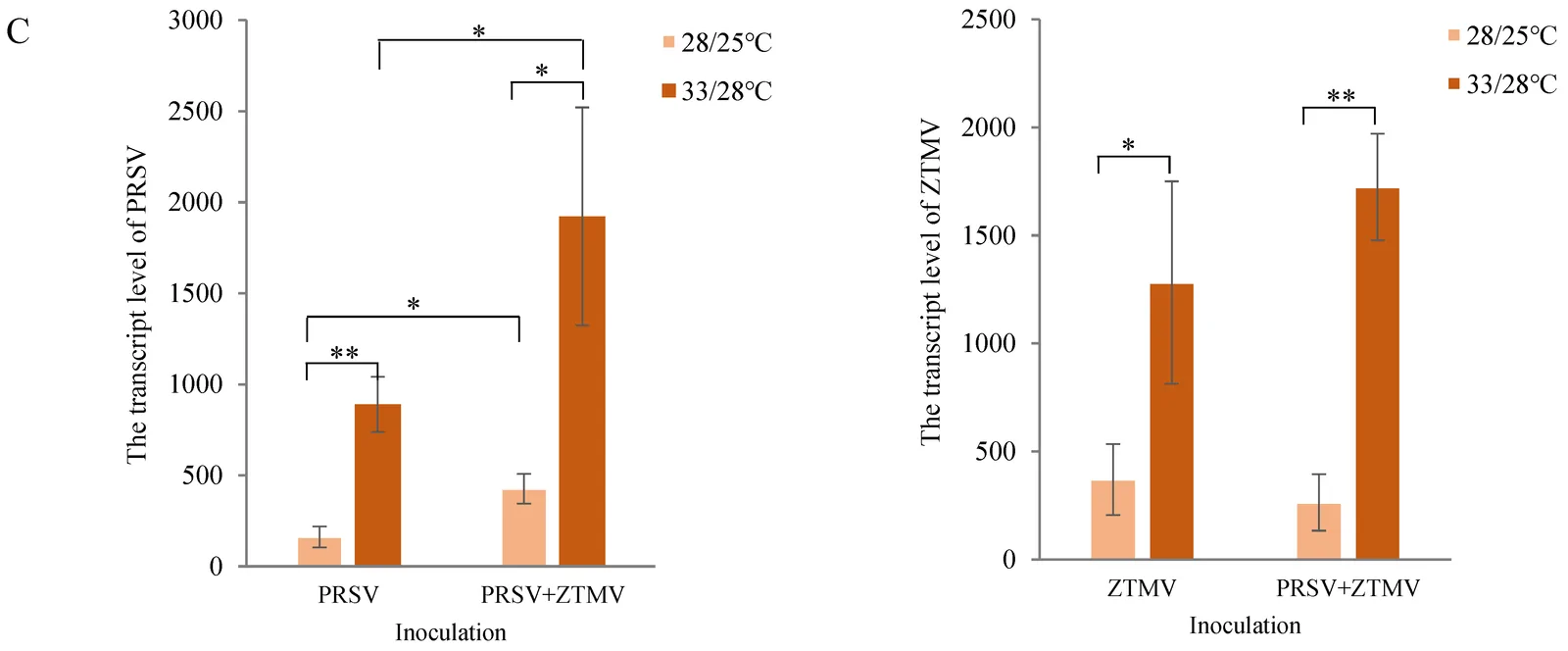
Symptom expression in watermelon seedlings following inoculation with infectious clones of PRSV (**A**) or PRSV with ZTMV mixed infection (**B**) under different temperature conditions, and the comparison of virus accumulation (**C**). Data are means ± SD from three biological replicates. Statistical analysis was conducted using Student’s *t* test to determine the significance. Significant differences between 33/28 °C and 28/25 °C are indicated: * *p* < 0.05 and ** *p* < 0.01. Bar: 1 cm.

To further clarify the causes of necrotic symptoms in watermelon plants infected by PRSV and ZTMV, we utilized the PRSV and ZTMV infectious clone that was constructed to inoculate watermelon seedlings. Six days post-inoculation, all plants exhibited mosaic symptoms. Subsequently, a half of plants from each inoculation treatment were transferred to high-temperature conditions of 28/33 °C for growth, while the remaining plants continued to be grown under 28/25 °C conditions. Seven days later, all PRSV-containing inoculated plants (including PRSV inoculation, PRSV plus ZTMV co-inoculation) developed necrotic lesions under high temperature, whereas only mosaic and malformation were observed at 28/25 °C conditions (Figure 5A,B), indicating that high temperature (>28 °C) is a key factor inducing PRSV-mediated necrosis. ZTMV-inoculated plants predominantly showed mosaic and malformation symptoms under both temperature regimes, with mild necrotic spots observed on the first true leaf in only a few individuals. RT-qPCR analysis showed that PRSV and ZTMV accumulation in plants, whether under mixed or single inoculation, was significantly greater at 33/28 °C than at 28/25 °C. Under the same temperature conditions, PRSV accumulation was significantly higher in PRSV and ZTMV co-inoculated plants than in PRSV- inoculated plants, whereas ZTMV accumulation exhibited no significant difference between co- inoculated and ZTMV-inoculated plants (Figure 5C). These results indicate that high temperature could promote the multiplication of PRSV or ZTMV. Mixed infection with the two viruses may also enhance the accumulation of PRSV.

**Figure 6:**
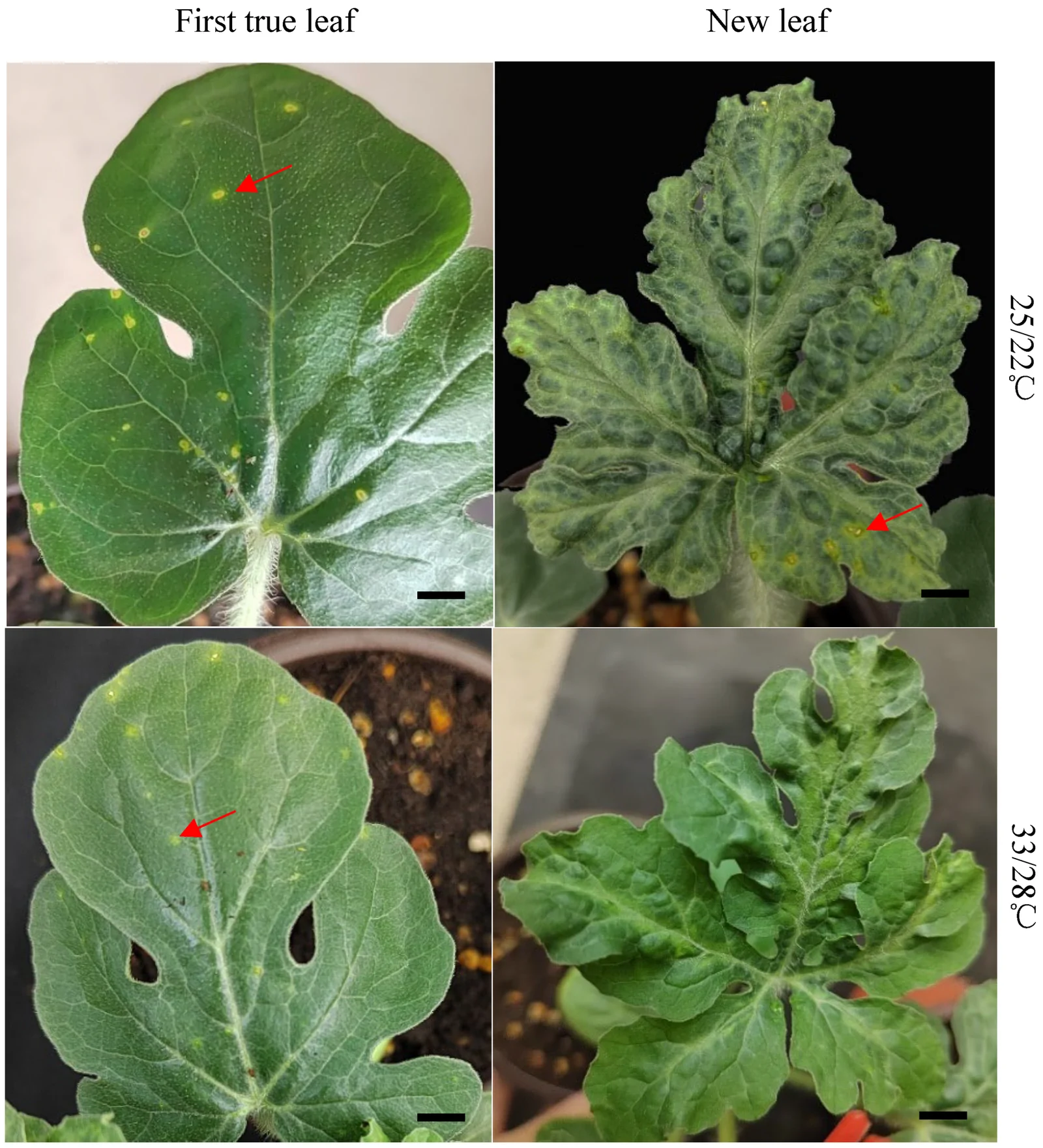
Symptom expression in watermelon seedlings following inoculation with infectious clones ZTMV under different temperature conditions. Bar: 0.5 cm. Red arrows indicate necrotic lesions.

Notably, at 28/25 °C, more plants exhibited necrosis (11/15) compared to those under 33/28 °C (6/15). When the 28/25 °C condition group was further transferred to low-temperature conditions (25/22 °C) for 3 days, necrosis also appeared on new leaves, whereas the high-temperature group retained mosaic and malformation symptoms (Figure 6) This suggests that low temperature (<25 °C) promotes the development of ZTMV-induced necrosis.

**Figure 7:**
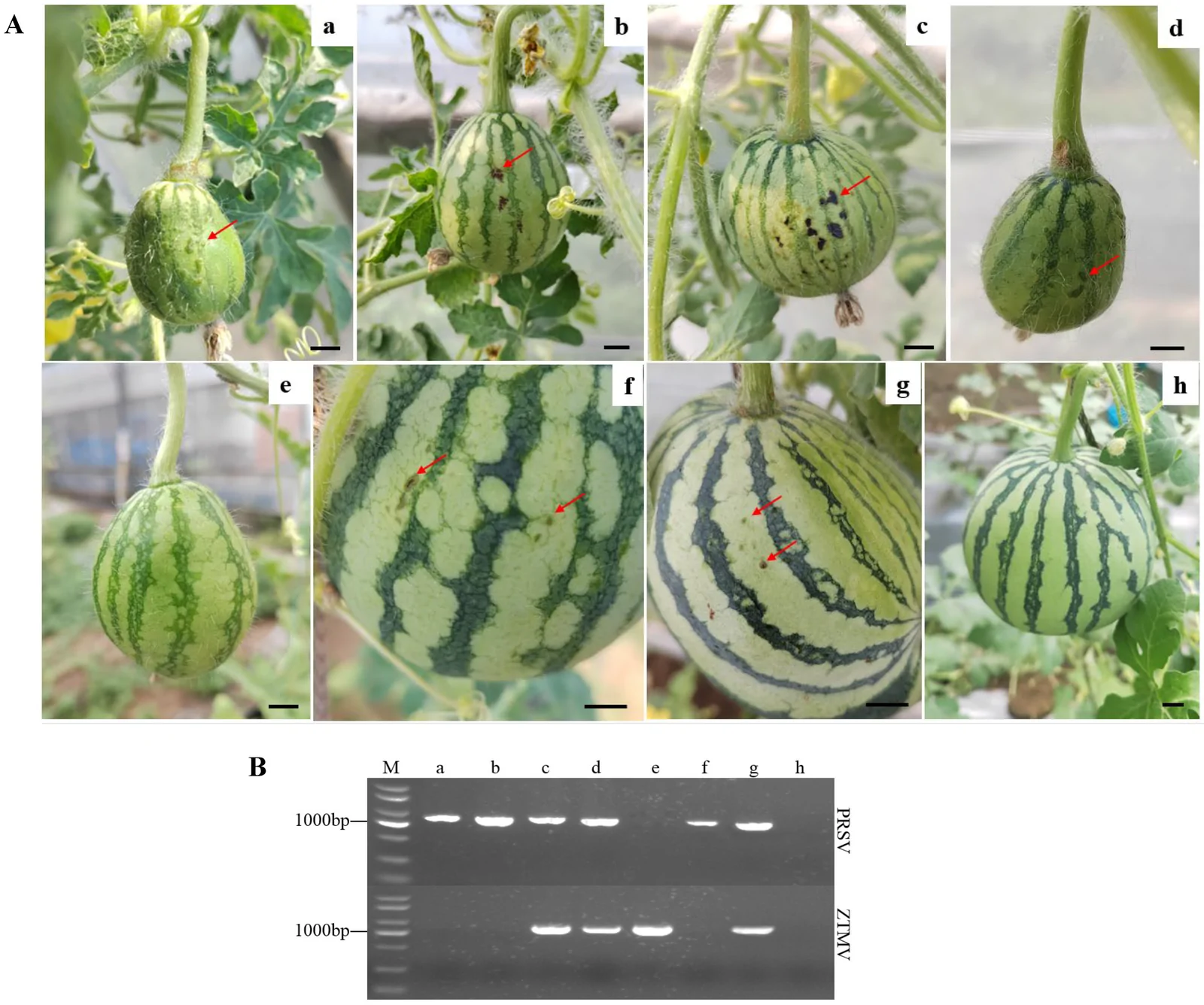
Symptom expression and RT-PCR results in virus- infected watermelon fruits. (**A**) Plants grown under greenhouse conditions were inoculated with infectious clones of PRSV alone (**a**,**b**), a mixture of PRSV and ZTMV infectious clones (**c**,**d**), ZTMV alone (**e**), sap extracted from XZW1 (**f**), or sap from XZW2 (**g**), and non-inoculated plants served as negative controls (**h**). (**B**) RT-PCR detection of PRSV and ZTMV in the inoculated plants shown in Figure 5A, using primer pairs ZT/PR-4819F with ZTMV-5790R or ZT/PR-4819F with PRSV1/2-5681R. Red arrow point to the symptoms of protrusions and/or necrotic lesions. Bar: 1 cm.

These virus-inoculated plants, including those inoculated with infectious clones or the sap of XZW1 and XZW2, were then transplanted to the field and artificially pollinated to set fruit (field temperature 25~40 °C). Plants inoculated with PRSV-containing inoculum developed fruit malformations characterized by epidermal protrusions and necrotic lesions, consistent with the “‘Mazi’ watermelon“ symptoms observed in the field. However, under the conditions of this experiment, fruits from plants inoculated exclusively with ZTMV did not display these symptoms. Molecular identification confirmed the presence of the respective viruses in the inoculated plants (Figure 7). These results fulfill the molecular Koch’s postulates for PRSV.

The integrated pathological analysis established PRSV as the primary etiological agent responsible for the characteristic “‘Mazi’ watermelon“ symptomatology, with ambient temperature serving as a critical modulating factor.

### 2.6. Local Temperature Conditions Favor Systemic Necrosis in Infected Plants

**Figure 8:**
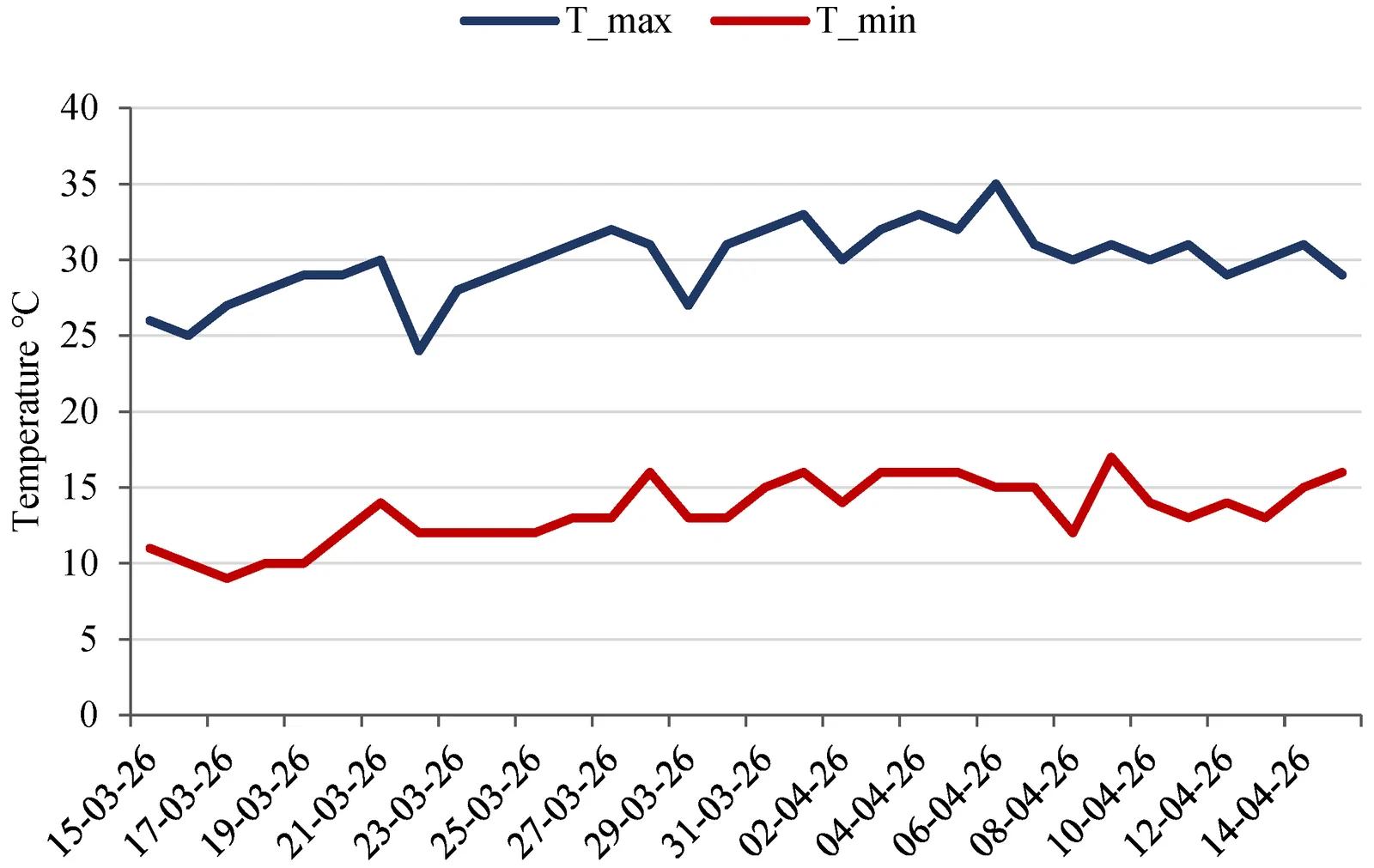
Temperature trends in Huaping County from 15 March to 15 April 2026 year.

In Huaping, watermelons are mainly sown in January, begin fruit setting in early March, and are harvested from mid-to-late April. We reviewed historical temperature records from 15 March to 15 April 2026 year, for the local area (Figure 8). The maximum temperatures ranged from 25 °C to 35 °C, with an average maximum of 30 °C. The minimum temperatures ranged from 9 °C to 17 °C, with an average minimum of 13 °C. Combined with the previous results, we can concluded that local temperature conditions, particularly the high-temperature periods, are the primary cause of systemic necrosis and severe fruit symptoms in infected plants under field conditions.

## 3. Discussion

### 3.1. PRSV Is the Primary Pathogen Causing “Mazi Watermelon” Symptoms in Huaping Watermelon

Through systematic pathogen isolation and identification, this study ruled out fungi and bacteria as the primary pathogens responsible for the “Mazi” symptoms in Huaping watermelon, ultimately confirming PRSV as the main causative agent. PRSV belongs to the genus *Potyvirus* in the family *Potyviridae* and primarily infects papaya and cucurbit crops. It is classified into two strains based on the host range: PRSV-P (infecting papaya and cucurbits) and PRSV-W (infecting only cucurbits) [7,8]. The PRSV isolates obtained from watermelon in this study belong to the PRSV-W strain, which is the predominant strain infecting cucurbit crops worldwide and can cause significant yield losses [9,10,11,12]. This classification is supported by our mechanical inoculation assays, in which papaya plants inoculated with the same viral inocula (from XZW1 and XZW2) showed no symptoms at 15 dpi, and RT-PCR failed to detect PRSV in these papaya samples, confirming the inability of this isolate to infect papaya. Previous reports indicate that PRSV infection in watermelon typically manifests as mosaic and malformation symptoms, with no documented cases of systemic necrosis [13].

ZTMV, also a member of *Potyvirus*, is closely related to PRSV [14]. Both viruses are transmitted by aphids in a non-persistent manner and induce similar symptoms in cucurbit crops. In this study, XZW-2 showed mixed infection with PRSV and ZTMV, while XZW-1 tested positive only for PRSV. Both plants exhibited similar “Mazi” watermelon symptoms, confirming PRSV as the primary pathogen responsible for the symptoms, with ZTMV potentially modulating symptom expression.

### 3.2. Temperature Is a Key Regulatory Factor in PRSV-Induced Necrosis in Watermelon

The occurrence of systemic necrosis following viral infection depends on multiple factors, including host genotype, viral strain, and environmental conditions [15]. Ambient temperature significantly modulates viral symptom expression [16,17,18,19]. High temperatures can exacerbate symptoms. For example, groundnut bud necrosis virus (GBNV) in cowpea promotes severe necrosis and viral RNA accumulation at 30 °C, while symptoms and systemic spread are suppressed at 15 °C [20]. This study found that PRSV induced severe systemic necrosis in watermelon at temperatures above 28 °C (28/33 °C), with young fruits developing necrotic spots. However, at temperatures exceeding 35 °C, necrotic spots ceased to expand, likely due to the “heat masking” effect, as observed with turnip mosaic virus (TuMV) in Chinese cabbage, where symptoms peaked at 18–28 °C but were mild at 33 °C [21]. Mangrauthia et al. [22] reported that PRSV symptoms and viral accumulation in papaya peaked within the optimal growth temperature range (26–31 °C) but declined significantly at temperatures 10 °C above or below this range.

Systemic necrosis results from a compatible interaction between the host and virus, characterized by virus-induced systemic programmed cell death, the severity of which is influenced by host genetic background and viral strain. For example, different soybean cultivars exhibit varying responses to the same isolate of soybean mosaic virus (SMV), classified as resistant (R, asymptomatic), necrotic (N, systemic necrosis), or susceptible (S, mosaic), indicating that necrosis is not solely determined by host susceptibility but by specific host–virus interactions [16,23]. Differences in viral strain also play a critical role in symptom variation. Distinct isolates of the same virus can induce markedly different symptoms in the same host. For instance, the EU-tom strain of pepino mosaic virus (PepMV) induces mild systemic mosaic in *Nicotiana occidentalis* 37B, whereas the Ch2 isolate PCH06/104 causes necrotic local lesions and severe systemic mosaic in the same host, readily differentiating these strains [15]. Similarly, the PVY^N^ strain of potato virus Y (PVY) causes systemic necrosis in tobacco, while the PVY^O^ strain results only in mild mosaic symptoms that diminish as the plant grows [24]. Although all three PRSV isolates we used in the inoculation experiments in this study (PRSV-HP1, -HP2, and -HaNHK10) induced necrosis, further in-depth and systematic investigation is required to establish whether the necrotic response is independent of the viral isolate requires. Unlike PRSV, ZTMV could induce mild systemic necrosis in watermelon below 25 °C (22/25 °C). Similarly, alternanthera mosaic virus (AltMV) caused only mosaic and local necrosis in *Nicotiana benthamiana* at 25 °C but triggered systemic necrosis and plant death at 15 °C [25], suggesting that low temperatures can also promote necrosis in certain host–virus interactions.

Combined with field conditions, the fruit development period of watermelons in Huaping County, Yunnan Province, is concentrated from late March to mid-April. During this period, the average daily maximum temperature is approximately 30 °C, which falls precisely within the optimal temperature range for PRSV-induced systemic necrosis. Meanwhile, the average nightly minimum temperature is around 13 °C, meeting the low-temperature conditions required for ZTMV-induced necrosis. If ZTMV infection occurs, the necrotic symptoms may also develop. This temperature range also favors aphid reproduction and migration, potentially accelerating the spread of PRSV and other aphid-transmitted viruses in the field and increasing the likelihood of mixed infection and systemic necrosis.

## 4. Materials and Methods

### 4.1. Sample Collection

In April 2026, two watermelon plants exhibiting typical “Mazi watermelon” symptoms were collected from fields in Xinzhuang Village, Huaping County, and designated as XZW1 and XZW2. Tissues including leaves, stems, and fruits were sampled for subsequent pathogen isolation and identification.

### 4.2. Pathogen Isolation and Purification

#### 4.2.1. Isolation, Culture, and Identification of Fungal Pathogens

The conventional tissue isolation method was employed as described previously [26]. Tissue blocks (5 mm × 5 mm) were excised from the margins of lesions on leaves, stems, and fruits. Surface sterilization was performed with 75% ethanol (15 s) and 2% sodium hypochlorite (4 min), followed by three sterile water rinses. Tissue samples were placed on potato dextrose agar (PDA) plates and incubated at 28 °C for 3~5 d. Mycelial tips emerging from the tissue blocks were transferred to fresh PDA plates for further culture and purified via single-spore isolation. Colony and spore morphologies were observed, and pathogenicity was verified by inoculating healthy watermelon leaves with mycelial plugs using an in vitro inoculation method.

#### 4.2.2. Isolation, Culture, and Identification of Bacterial Pathogens

The spread plate method was used [27]. Lesion tissues were surface-sterilized, ground in sterile water to prepare a suspension, and serially diluted. Then 100 μL aliquots were spread onto LB agar plates, followed by incubation at 28 °C for 3~5 d. Single colonies with different morphologies were transferred to LB liquid medium for propagation. The isolates were subjected to 16S rDNA amplification and sequencing. Bacterial suspensions were inoculated onto healthy watermelon seedlings via spray inoculation for pathogenicity testing.

#### 4.2.3. Virus Identification

Small RNA sequencing [28]. Total RNA was extracted from XZW1 and XZW2, respectively, and then mixed in equal amounts as the sample for small RNA sequencing. The mixed sample was sent to Sangon Biotech (Shanghai) Co., Ltd. for small RNA library construction, high-throughput sequencing and analysis. After sequencing, raw reads were processed to remove adapter sequences, reads shorter than 10 nt or longer than 45 nt, and low-quality reads to obtain clean reads. De novo assembly of the clean reads was performed using SPAdes v3.15.5 [29] with a k-mer size of 15 to generate contigs. The assembled contigs were subsequently aligned against the watermelon reference genome (*Citrullus lanatus*, available at http://cucurbitgenomics.org/organism/21, accessed on 25 May 2026) to filter out host-derived sequences. The remaining unmapped contigs were then subjected to BLASTn analysis against the NCBI nucleotide database (nt) to identify virus species and obtain viral sequence information present in the sample.

Molecular detection. Total RNA was extracted from diseased plant leaves using the RNAsimple Total RNA Kit (Tiangen, Beijing, China). First-strand cDNA was synthesized from 1 μg of total RNA using the FastKing RT Kit (Tiangen, Beijing, China) and PCR was subsequently performed. The PCR protocol was as follows: initial denaturation at 95 °C for 3 min; 38 cycles of 95 °C for 10 s, 60 °C for 10 s, and 72 °C for 30–90 s; and a final extension at 72 °C for 5 min. Based on the small RNA sequencing results, specific primers for PRSV and ZTMV (Table S1). RT-PCR was conducted on the field-collected diseased plants and inoculated watermelon seedlings inoculated with sap from diseased plants and infectious clones to verify viral infection.

### 4.3. Artificial Inoculation

Mechanical inoculation. Pericarp tissue from diseased fruits was ground in 0.1 M PBS buffer (pH 7.0). The homogenate was mechanically inoculated onto 30 healthy watermelon seedlings of *Citrullus lanatus* cv. Meidu. Fifteen seedlings were placed in a growth chamber (16 h light/8 h dark, 28 °C/25 °C), and the other fifteen were placed in a greenhouse (daytime 25~35 °C, nighttime 20~25 °C). Disease development was observed 7–15 days post-inoculation.

Infectious clone inoculation. Infectious clones of PRSV-HaNHK10 (OK465456) and ZTMV-CX1 (MK988416) isolates constructed by our team [30,31], were used to inoculate 30 healthy watermelon seedlings each via *Agrobacterium* infiltration. Disease development was observed 7~15 days post-inoculation. All inoculated plants were initially cultured in a growth chamber (day/night: 28 °C/25 °C). When typical viral symptoms such as mosaic appeared, a subset of symptomatic plants from each treatment was transferred to high-temperature conditions (day/night: 33 °C/28 °C) for an additional 10 days, while the remaining plants continued culture at normal temperature (28 °C/25 °C) to observe symptom differences between the two temperature regimes. Finally, the differently inoculated plants were transplanted to the field (25~40 °C) and allowed to set fruit through artificial pollination to observe fruit symptoms.

### 4.4. RT-qPCR

Young leaf samples were collected at 14 days post inoculation to analyze the accumulation of virus by quantitative real-time PCR. It was performed in a LightCycler 480 instrument using Taq Pro Universal SYBR qPCR Master Mix (Vazyme, Nanjing, China). Following heating of the template to 95 °C for 30 s, the cycling conditions were 95 °C for 10 s, 60 °C for 15 s, 72 °C for 20 s, and fluorescence acquisition at 72 °C. The *tubulin* gene (*Cla97C02G050600*) served as the internal control [32]. The viral relative RNA levels were determined via the 2^−ΔCt^ method with three biological replicates. The specific primers for RT-qPCR are shown in Table S1. The experiment was repeated twice with three biological replicates. Statistical analysis was conducted using Student’s *t* test to determine the significance.

## 5. Conclusions

This study identified the primary pathogen responsible for the “Mazi watermelon” disease in watermelons in Huaping County, Yunnan Province. Through small RNA sequencing and artificial inoculation experiments, we confirmed that PRSV is the key pathogen inducing typical symptoms of “Mazi watermelon”. Temperature significantly regulates viral pathogenicity. Under high-temperature conditions (>28 °C), PRSV can trigger systemic necrosis in watermelons. This study is the first to elucidate the etiological basis of PRSV infection causing “’Mazi’ watermelon” and reveals the mechanism by which temperature modulates virus–host interactions to determine the severity of necrotic symptoms, providing a scientific foundation for disease monitoring, early warning, and integrated control strategies.

## Data Availability

The dataset supporting the conclusions of this article is available in the NCBI SRA under accession number PRJNA1534151, https://www.ncbi.nlm.nih.gov/sra/PRJNA1534151, submitted on 24 September 2026.

## Supplementary Materials

The following supporting information can be downloaded at: https://www.mdpi.com/article/10.3390/ijms27219895/s1.

## References

[1] Kwon O.K., Jeong A.R., Jeong Y.J., Kim Y.A., Shim J., Jang Y.J., Lee G.P., Park C.J. (2021). Incidence of Alternaria Species Associated with Watermelon Leaf Blight in Korea. Plant Pathol. J..

[2] Ma G., Bao S., Zhao J., Sui Y., Wu X. (2021). Morphological and Molecular Characterization of Alternaria Species Causing Leaf Blight on Watermelon in China. Plant Dis..

[3] Wang H., You S., Yang M., Shao J., Jin B. (2025). Whole genome sequence of *Cercospora citrullina* strain UNL090101, the causal agent of watermelon spot disease. Microbiol. Resour. Announc..

[4] Latin R.X., Hopkins D.L. (1995). Bacterial Fruit Blotch of Watermelon—The Hypothetical Exam Question Becomes Reality. Plant Dis..

[5] Kwak H.R., Kim J.S., Cho J.D., Lee J.H., Kim T.S., Kim M.K., Choi H.S. (2015). Characterization of Melon necrotic spot virus Occurring on Watermelon in Korea. Plant Pathol. J..

[6] Katoh K., Rozewicki J., Yamada K.D. (2019). MAFFT online service: Multiple sequence alignment, interactive sequence choice and visualization. Brief. Bioinform..

[7] Desbiez C., Lecoq H., Bamford D.H., Zuckerman M. (2021). Papaya Ringspot Virus (*Potyviridae*). Encyclopedia of Virology.

[8] Gonsalves D., Suzuki J.Y., Tripathi S., Ferreira S.A., Mahy B., Van Regenmortel M. (2008). Papaya Ringspot Virus. Encyclopedia of Virology.

[9] Guner N., Strange E.B., Wehner T.C., Pesic-VanEsbroeck Z. (2002). Methods for screening watermelon for resistance to papaya ringspot virus type-W. Sci. Hortic..

[10] Yu T.A., Chiang C.H., Wu H.W., Li C.M., Yang C.F., Chen J.H., Chen Y.W., Yeh S.D. (2011). Generation of transgenic watermelon resistant to Zucchini yellow mosaic virus and Papaya ringspot virus type W. Plant Cell Rep..

[11] Mansilla P.J., Moreira A.G., Mello A.P.O.A., Rezende J.A.M., Ventura J.A., Yuki V.A., Levatti F.J. (2013). Importance of cucurbits in the epidemiology of Papaya ringspot virus type P. Plant Pathol..

[12] Sun Y., Lu J., Tang G., Zhang Q., Gao W., Li W. (2018). First Detection of Papaya Ringspot Virus on *Cucurbita pepo* in Wenshan State of Yunnan Province. Plant Prot..

[13] Lecoq H., Desbiez C. (2012). Viruses of cucurbit crops in the Mediterranean region: An ever-changing picture. Adv. Virus Res..

[14] Desbiez C., Wipf-Scheibel C., Lecoq H. (2014). Zucchini tigré mosaic virus is a distinct potyvirus in the papaya ringspot virus cluster: Molecular and biological insights. Arch. Virol..

[15] Blystad D.R., van der Vlugt R., Alfaro-Fernández A., Córdoba M.D., Bese G., Hristova D., Pospieszny H., Mehle N., Ravnikar M., Tomassoli L. (2015). Host range and symptomatology of strains occurring in Europe. Eur. J. Plant Pathol..

[16] Zheng C., Chen P., Gergerich R. (2005). Effect of Temperature on the Expression of Necrosis in Soybean Infected with Soybean mosaic virus. Crop Sci..

[17] Li D.X., Chen P.Y., Shi A.N., Shakiba E., Gergerich R., Chen Y.F. (2009). Temperature Affects Expression of Symptoms Induced by Soybean Mosaic Virus in Homozygous and Heterozygous Plants. J. Hered..

[18] Feng X., Poplawsky A.R., Karasev A.V. (2014). A Recombinant of Bean common mosaic virus Induces Temperature-Insensitive Necrosis in an I Gene-Bearing Line of Common Bean. Phytopathology.

[19] Tsai W.A., Shafiei-Peters J.R., Mitter N., Dietzgen R.G. (2022). Effects of Elevated Temperature on the Susceptibility of Capsicum Plants to Capsicum Chlorosis Virus Infection. Pathogens.

[20] Singh A., Permar V., Basavaraj, Tomar B.S., Praveen S. (2018). Effect of Temperature on Symptoms Expression and Viral RNA Accumulation in Groundnut Bud Necrosis Virus Infected *Vigna unguiculata*. Iran. J. Biotechnol..

[21] Chung B.N., Choi K.S., Ahn J.J., Joa J.H., Do K.S., Park K.S. (2015). Effects of Temperature on Systemic Infection and Symptom Expression of Turnip mosaic virus in Chinese cabbage (*Brassica campestris*). Plant Pathol. J..

[22] Mangrauthia S.K., Singh Shakya V.P., Jain R.K., Praveen S. (2009). Ambient temperature perception in papaya for papaya ringspot virus interaction. Virus Genes.

[23] Usovsky M., Chen P., Li D., Wang A., Shi A., Zheng C., Shakiba E., Lee D., Canella Vieira C., Lee Y.C. (2022). Decades of Genetic Research on Soybean mosaic virus Resistance in Soybean. Viruses.

[24] Quenouille J., Vassilakos N., Moury B. (2013). Potato virus Y: A major crop pathogen that has provided major insights into the evolution of viral pathogenicity. Mol. Plant Pathol..

[25] Lim H.S., Nam J., Seo E.Y., Nam M., Vaira A.M., Bae H., Jang C.Y., Lee C.H., Kim H.G., Roh M. (2014). The coat protein of Alternanthera mosaic virus is the elicitor of a temperature-sensitive systemic necrosis in *Nicotiana benthamiana*, and interacts with a host boron transporter protein. Virology.

[26] Guo Z., Luo C.X., Wu H.J., Peng B., Kang B.S., Liu L.M., Zhang M., Gu Q.S. (2022). Colletotrichum Species Associated with Anthracnose Disease of Watermelon (*Citrullus lanatus*) in China. J. Fungi.

[27] Peng S., Ren Y., Yao T., Chu H., Gao Y., Tian X., Zhang Y. (2023). First Report of *Pseudomonas palleroniana* Causing Potato Soft Rot in China. Plant Dis..

[28] Pooggin M.M. (2018). Small RNA-Omics for Plant Virus Identification, Virome Reconstruction, and Antiviral Defense Characterization. Front. Microbiol..

[29] Bankevich A., Nurk S., Antipov D., Gurevich A.A., Dvorkin M., Kulikov A.S., Lesin V.M., Nikolenko S.I., Pham S., Prjibelski A.D. (2012). SPAdes: A new genome assembly algorithm and its applications to single-cell sequencing. J. Comput. Biol..

[30] Liu L., Peng B., Kang B., Wu H., Liu X., Gu Q. (2024). The genome and infectious clone of papaya ringspot virus melon isolate. China Cucurbits Veg..

[31] Peng B., Liu L., Wu H., Kang B., Fei Z., Gu Q. (2021). Interspecific Recombination Between Zucchini Tigre Mosaic Virus and Papaya Ringspot Virus Infecting Cucurbits in China. Front. Microbiol..

[32] Hao X., Liu F., Liu L., Wu H., Liang Z., Zhao W., Wang Y., Gu Q., Kang B. (2024). Zucchini yellow mosaic virus-induced hypersensitive response is associated with pathogenesis-related 1 protein expression and confers resistance in watermelon. Plant Cell Rep..

