# Supplementary material for: High Temperature Triggers Systemic Necrosis and Reveals PRSV as the Primary Cause of “‘Mazi’ Watermelon”
Source: Int J Mol Sci. 2026 Nov 4;27(21):9895. doi: 10.3390/ijms27219895 (PMC13764386; doi:10.3390/ijms27219895)
Supplement: Supplementary file 1 [file ijms-27-09895-s001.zip › ijms-4570786-supplementary.pdf]

Table S1. RT-PCR Primer Information for PRSV and ZTMV

Primer	Sequence (5′-3′)	Tm (℃)	Amplicon sizes (bp)	Usage
P-F1	GTTCATTTCATTTGGAGAGGAAAAA TAAAACATCTCAACACAACAC	71.5	3590	amplification for full genome of PRSV
P-R1	CACGCCAYTCTTCCTGCAA	54		
P-F2	TTGCAGGAAGARTGGCGTG	56	3415	
P-R2	CAAACCACRGAAGCTATRCC	56		
P-F3	GGYATAGCTTCYGTGGTTTG	58	2002	
P-R3	CCCCAKGAYTCTATCATTGC	57		
P-F4	GTTGCCATTGGATATCCACGAATG	59	1387	
P-R4	CTCATGATCAGGATGAATGGCGA	57		
P-F5	TAATGGTGATGATCTCTGTATCGCCA	58	1600	
P-R5	GAAGATTTGAACACACGTGGGACAC	59		
Z-F1	CATTTCCACAACTTGCAATCATGTC	56	2700	amplification for full genome of ZTMV
Z-R1	CGCTTCTCCTTCAGGTATGTTCACA	61		
Z-F2	CGCTTTCGTCAGCAAATGGTTG	58	3313	
Z-R2	GCTTGCCAAATCTGTTCATACACCC	59		
Z-F3	GCTGCGTGATAGCGAAATCAAGC	58	3248	
Z-R3	CGTTCAGGTTCGAGCTTTGGAATG	61		
Z-F4	GCAGGCTACGACCGTGAGAGG	60	1790	
Z-R4	GAAGATTTGAACACACGTGGGACAC	59		
ZT/PR-4819F	CCRCCTGGGCGYGAAGTTGA	58		detection
ZTMV-5790R	GGGACATCACGCACATAAAAAGG	57	970	
PRSV1/2-5681R	AASGGTATTCGMACATCRYYYTCC	60	860	
PRSV-qF	GGAAAAGTGCCGCGAAAGGTT	59	255	RT-qPCR
PRSV-qR	TCACGCTCCATCACGAAATCAAG	59		
ZTMV-qF	AAGACAAACAGGGCCTGCTAAGC	58	137	
ZTMV-qR	GGAGATGACAAACGACAGAAGCG	58		

*: Sequence positions may vary slightly due to differences in the 5′ utr length.

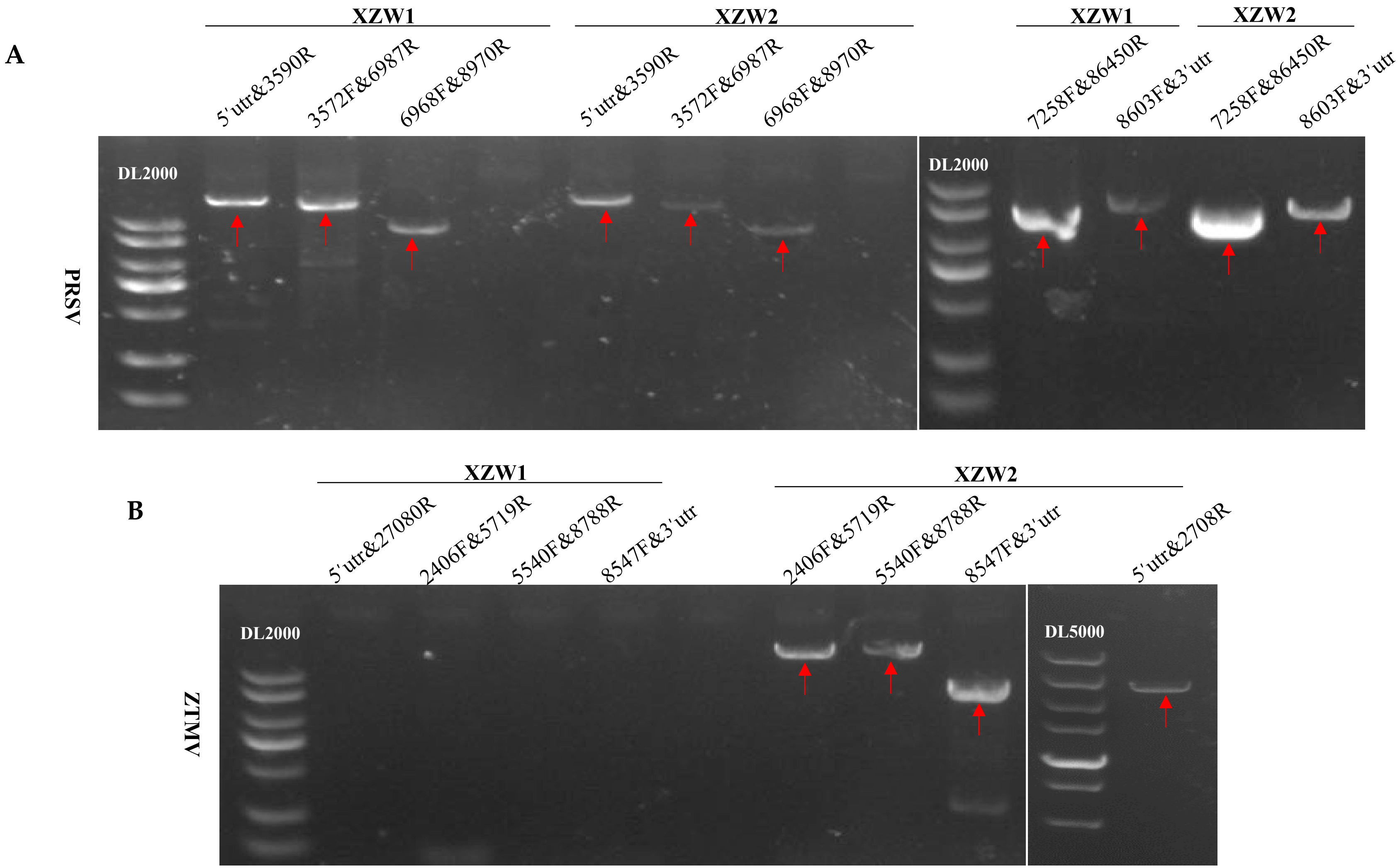

Figure S1. Electrophoresis of amplified fragments of the PRSV and ZTMV. (A) Fragments covering the complete genomes of two PRSV isolates amplified by RT-PCR using five pairs of primers. (B) Fragments covering the complete genomes of ZTMV isolate, amplified by RT-PCR using four pairs of primer.
